# Pegylated Interferon Treatment for the Effective Clearance of Hepatitis B Surface Antigen in Inactive HBsAg Carriers: A Meta-Analysis

**DOI:** 10.3389/fimmu.2021.779347

**Published:** 2021-11-04

**Authors:** Aixin Song, Xiao Lin, Junfeng Lu, Shan Ren, Zhenhuan Cao, Sujun Zheng, Zhongjie Hu, Hong Li, Chengli Shen, Xinyue Chen

**Affiliations:** ^1^ First Department of Liver Disease Center, Beijing Youan Hospital, Capital Medical University, Beijing, China; ^2^ Division of Surgical Oncology, James Cancer Hospital, The Ohio State University Wexner Medical Center, Columbus, OH, United States

**Keywords:** HBV, HBsAg, high clearance rate, IHC, meta-analysis

## Abstract

**Background:**

Expanding antiviral therapy to benefit more populations and optimizing treatment to improve prognoses are two main objectives in current guidelines on antiviral therapy. However, the guidelines do not recommend antiviral therapy for inactive hepatitis B surface antigen (HBsAg) carriers (IHCs). Recent studies have shown that antiviral therapy is effective with good treatment outcomes in IHC populations. We conducted a systematic review and meta-analysis of HBsAg clearance and conversion in IHCs.

**Methods:**

We searched PubMed, Embase, Medline, and Web of Science to retrieve articles on HBsAg clearance in IHCs published between January 2000 and August 2021. Data were collected and analysed using the random-effects model for meta-analysis.

**Results:**

A total of 1029 IHCs from 11 studies were included in this analysis. The overall HBsAg clearance rate was 47% (95% confidence interval (CI): 31% - 64%), with a conversion rate of 26% (95% CI: 15% - 38%) after 48 weeks of Pegylated interferon (Peg-IFN) treatment. In the control group (including nucleos(t)ide analogue (NA) treatment or no treatment), the overall HBsAg clearance rate was only 1.54% (95% CI: 0.56% - 3.00%), which was markedly lower than that in the Peg-IFN group. Further analysis showed that a low baseline HBsAg level and long treatment duration contributed to a higher HBsAg clearance rate.

**Conclusion:**

This study showed that treatment of IHCs can be considered to achieve a clinical cure for chronic hepatitis B virus (HBV) infection. After Peg-IFN treatment, the HBsAg clearance rate was 47%, and the conversion rate was 26%, which are markedly higher than those reported by previous studies on Peg-IFN treatment in patients with chronic hepatitis B (CHB). A low baseline HBsAg level and long treatment duration were associated with HBsAg clearance in IHCs. Therefore, antiviral therapy is applicable for IHCs, a population who may be clinically cured.

**Systematic Review Registration:**

http://www.crd.york.ac.uk/PROSPERO, CRD): CRD42021259889.

## Introduction

Chronic hepatitis B virus (HBV) infection is an important cause of liver cirrhosis and hepatocellular carcinoma (HCC) (1). To reduce this major threat, two main trends are evident in current Chinese and international guidelines on antiviral therapy for the prevention and treatment of chronic hepatitis B (CHB): 1) the use of de-escalation therapy by relaxing the treatment criteria, allowing more patients to receive treatment and thus improving the prognosis; and 2) optimization of clinical clearance and treatment outcomes in the appropriate populations. However, the guidelines do not recommend antiviral therapy for inactive hepatitis B surface antigen (HBsAg) carriers (IHCs) (2–4), whereas studies (5, 6) in Asian populations have suggested that treating IHC patients is important, mainly because Asian patients usually have a long disease course by the IHC stage, and a risk of developing cirrhosis and HCC remains without treatment.

Immune function can control HBV DNA and maintain HBsAg at a low level (close to a clinical functional cure) in IHCs. Moreover, the cumulative number of patients receiving antiviral therapy has increased over the past decades, and the population of patients with low HBsAg levels has expanded significantly. In the past, few studies have focused on whether IHCs are eligible for antiviral therapy and how such therapy may benefit patients. In this study, we performed a literature review and meta-analysis of recent reports.

## Methods

This review was registered in the International Prospective Register of Systematic Reviews; the protocol is available online (PROSPERO, http://www.crd.york.ac.uk/PROSPERO, CRD): CRD42021259889).

### Data Sources and Search Strategy

We followed the Preferred Reporting Items for Systematic Reviews and Meta-Analyses (PRISMA) guidelines (7) and conducted a search for studies reporting HBsAg clearance and conversion in IHC patients with low HBsAg levels. We searched PubMed, Embase, Medline, and Web of Science to retrieve relevant articles published in the past 20 years, i.e., from January 2000 to August 2021. The keywords included “hepatitis B virus”, “hepatitis B surface antigen”, “HBsAg”, “seroclearance or loss”, “seroconversion”, “clearance”, “undetectable”, “inactive hepatitis B surface antigen carrier”, “inactive chronic hepatitis B virus carrier”, “low surface antigen level”, “HBsAg level”, “peginterferon”, “pegylated-interferon”, “treatment”, “nucleos(t)ide analogues”, and “therapy”. The articles retrieved were further screened. Furthermore, all references included in the articles were manually searched to identify additional potentially eligible articles, and authors were contacted for more details if needed. The studies included randomized controlled trials (RCTs) and prospective or retrospective cohort studies published in English or Chinese without any geographical restrictions. Reviews, comments, letters, and case reports were excluded.

### Study Selection and Data Extraction

The titles, abstracts, and keywords of eligible articles were screened. Next, the abstracts and the full texts were carefully read for further screening, and duplicate publications were excluded. Two researchers independently completed this process and assessed the relevance of each study and the quality of the methodology. Any discrepancy was resolved with the help of a third researcher.

The inclusion criteria were as follows: (a) studies with more than 20 IHCs with HBsAg clearance data after pegylated interferon (Peg-IFN) treatment, nucleos(t)ide analogue (NA) treatment, or no treatment; and (b) studies with adequate data, including the frequency and rate of HBsAg clearance and observations with at least 24 weeks of Peg-IFN treatment, NA treatment, or no treatment. Studies with inadequate data and studies including patients with liver transplantation or HCC before HBsAg clearance or with human immunodeficiency virus (HIV), hepatitis C virus (HCV), or hepatitis D virus (HDV) coinfection were excluded.

Two researchers independently extracted data from the articles with a standard form. Data collected included the author, year of publication, country/region where the study was conducted, study design, sample sizes of the Peg-IFN group and the control group, patient ages, and baseline HBV DNA levels. Outcome data included the frequency and rate of HBsAg clearance and HBsAg conversion and Peg-IFN treatment courses in different groups.

### Outcomes and Definitions

In this meta-analysis, IHC status was defined as HBsAg(+) >6 months, HBsAg <1500 IU/mL, HBeAg (–), anti-HBe(+) or (-), anti-HBc(+), HBV DNA < 2000 IU/mL, normal alanine transaminase (ALT), and no cirrhosis on ultrasound or FibroScan. The treatment group received Peg-IFN, and the control group received NA or no treatment. The outcome measures were HBsAg clearance and conversion after 48 weeks of treatment or follow-up. In addition, subgroup analyses were performed to evaluate the correlations between baseline HBsAg and treatment course and HBsAg clearance.

### Assessment of Evidence Quality

Two researchers independently assessed the quality of the articles. The Newcastle-Ottawa Scale (NOS) was used to assess quality and bias (8). The NOS scale has three general areas consisting of eight items, including the selection of study groups, comparability of groups, and assessment of outcomes. The total score is 9; scores of 7 or higher indicate excellent quality, scores from 4 to 6 indicate fair quality, and scores of 4 or lower indicate poor quality (**Table S1**).

### Statistical Analysis

The main statistic was rates. A random-effects model was used to summarize HBsAg clearance and conversion rates. Because the rates of outcome measures in the control group may be close to zero or 100 (if not zero), Freeman-Tukey double-arcsine transformation was used to stabilize the variance, and the Wilson method was used to calculate the 95% confidence intervals (CIs) (9). Finally, the values were reverse-transformed for visualization in figures. The *I^2^* test and Cochrane’s *Q* test were performed to assess among-study heterogeneity, and a funnel plot and Egger’s test were used to assess any publication bias (10). Stata v14.0 was used for the data analysis, *P* values were two-tailed, and *P*<0.05 was considered statistically significant.

## Results

**Figure 1:**
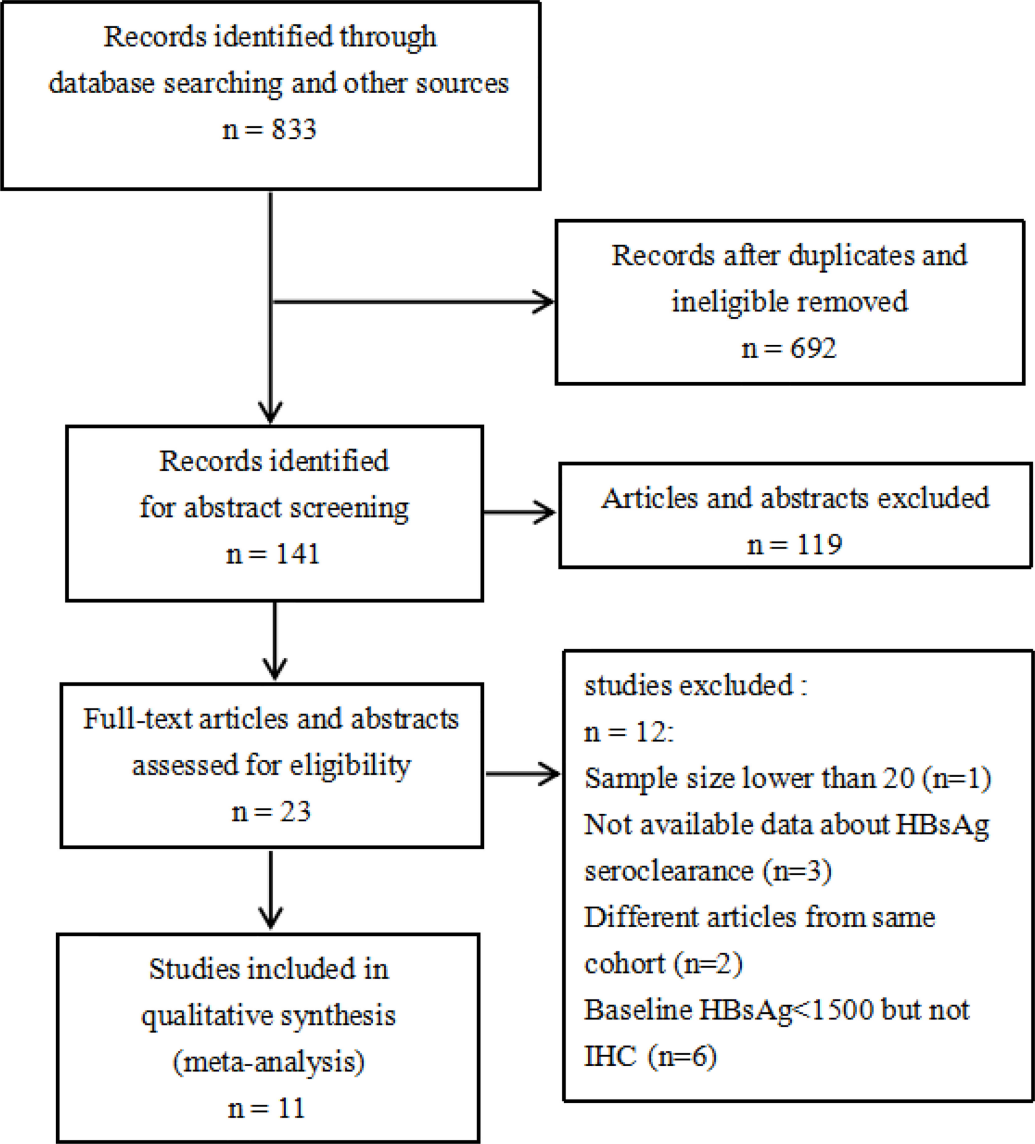
Study selection process.

We initially retrieved a total of 833 articles (**Figure 1**), and 141 articles remained after excluding duplicate publications. After screening the abstracts and full texts, 23 articles remained. After final screening, 12 articles were excluded, i.e., one article with a small sample size, three articles with incomplete outcome data, two articles with the same cohort, and six articles with baseline HBsAg <1500 IU/mL but not in compliance with one or more IHC criteria. Finally, a total of 11 articles were included in this meta-analysis (11–21).

### Characteristics of the Studies

The characteristics of the included studies are shown in **Table 1**. All 11 studies were conducted in Asia, including 10 in China and one in Singapore (13). Seven articles were published in English (11–15, 20, 21) and four in Chinese (16–19). The baseline HBsAg levels were <20 IU/mL in two studies (14, 16), <100 IU/mL in one study (12), <1000 IU/mL in six studies (11, 13, 17, 18, 20, 21), and <1500 IU/mL in two studies (15, 19). Ten studies used Peg-IFN treatment, with NA treatment (n=1) or no treatment (n=9) in the control group. One study applied only Peg-IFN treatment, with no control group (19). Eight of the 11 studies reported HBsAg clearance and conversion rates among IHCs after Peg-IFN treatment, and three studies reported only HBsAg clearance rates (13, 17, 20).

**Table 1:** Characteristics of the included studies.

Study	Region	Design	HBsAg level (IU/mL)	Sample size				Treatment period	HBsAg clearance rate (IFN)	HBsAg conversion rate (IFN)	HBsAg clearance rate (control)	Age(mean or median)	HBV DNA(IU/mL)
				Total	IFN	NA	None						
Cao 2017 (11)	Beijing, China	Prospective	<1000	144	102	–	42	96 W	48 W: 29.8% (28/94) 96 W: 44.7% (42/94)	48 W: 20.2% (19/94) 96 W: 38.3% (36/94)	48 W: 2.5% (1/40) 96 W: 2.5% (1/40)	38.8±10.0* 39.8±10.6#	<2000
Li 2016 (12)	Beijing, China	Retrospective	<100	60	20	–	40	72 W	72 W: 60% (12/20) 96 W: 65% (13/20)	72 W: 55% (11/20) 96 W: 60% (12/20)	0	33.80±11.45* 33.85±8.37#	<100
Lim 2019 (13)	Singapore	RCT	<1000	90	60	–	30	24 W 48 W	24 W: 30% (9/30) 48 W: 20% (6/30)	–	0	50.09±10.18* 49.5±10.86#	<2000
Zeng 2020 (14)	Zhengzhou, China	Retrospective	<20	32	16	–	16	48 W	24 W: 68.8% (11/16) 48 W: 93.8% (15/16)	48 W: 31.2% (5/16)	0	34 (32~46.8)* 36 (32~44.8)#	undetectable or <200
Wu 2021 (15)	Xi'an, China	Retrospective	<1500	298	142	–	156	48 W	48 W: 43.3% (58/134) 72 W: 50.7% (68/134)	48 W: 29.9% (40/134) 72 W: 38.8% (52/134)	48 W: 1.4% (2/143) 72 W: 2.1% (3/143)	37.9±10.7* 36.6±10.6#	<2000
Zhao 2020 (16)	Xiamen, China	Retrospective	<15	38	12	–	26	24 W	24 W: 83.3% (10/12)	24 W: 41.6% (5/12)	55 W: 7.7% (2/26)	37.3* 35.9#	<20
Shi 2018 (17)	Jiangsu, China	Retrospective	<1000	80	40 (add-on)	40	–	48 W	24 W: 20% (8/40) 48 W: 32.5% (13/40)	–	24 W: 2.5% (1/40) 48 W: 6.3% (3/40)	34.6±3.2	<3.3lg copies/mL
Zhou 2020 (18)	Chongqing, China	Retrospective	<1000	107	77	–	30	96 W	48 W: 24.7% (19/77) 96 W: 40.3% (31/77)	48 W: 9.1% (7/77) 96 W: 19.5% (15/77)	0	41.3±9.5* 43.4±10.1#	undetectable or <200
Chen 2020 (19)	Jilin, China	Retrospective	<1500	51	51 (add-on for 3 months)	–	–	48 W	48 W: 37.25% (19/51)	48 W: 15.69% (8/51)	–	–	<20
Chen 2021 (20)	Sichuan, China	Retrospective	<1000	90	27	–	63	48 W	24 W: 40.7% (11/27) 48 W: 55.6% (15/27)	–	0	–	–
Huang 2021 (21)	Hunan, China	Retrospective	<1000	39	19	–	20	72 W	48 W: 84.2% (16/19)	48 W: 68.2% (13/19)	0	39.00±11.55* 39.8±8.03#	<2000

Age: * for the IFN group, # for the control group.

### Overall 48-Week HBsAg Clearance Rates in IHCs

**Figure 2:**
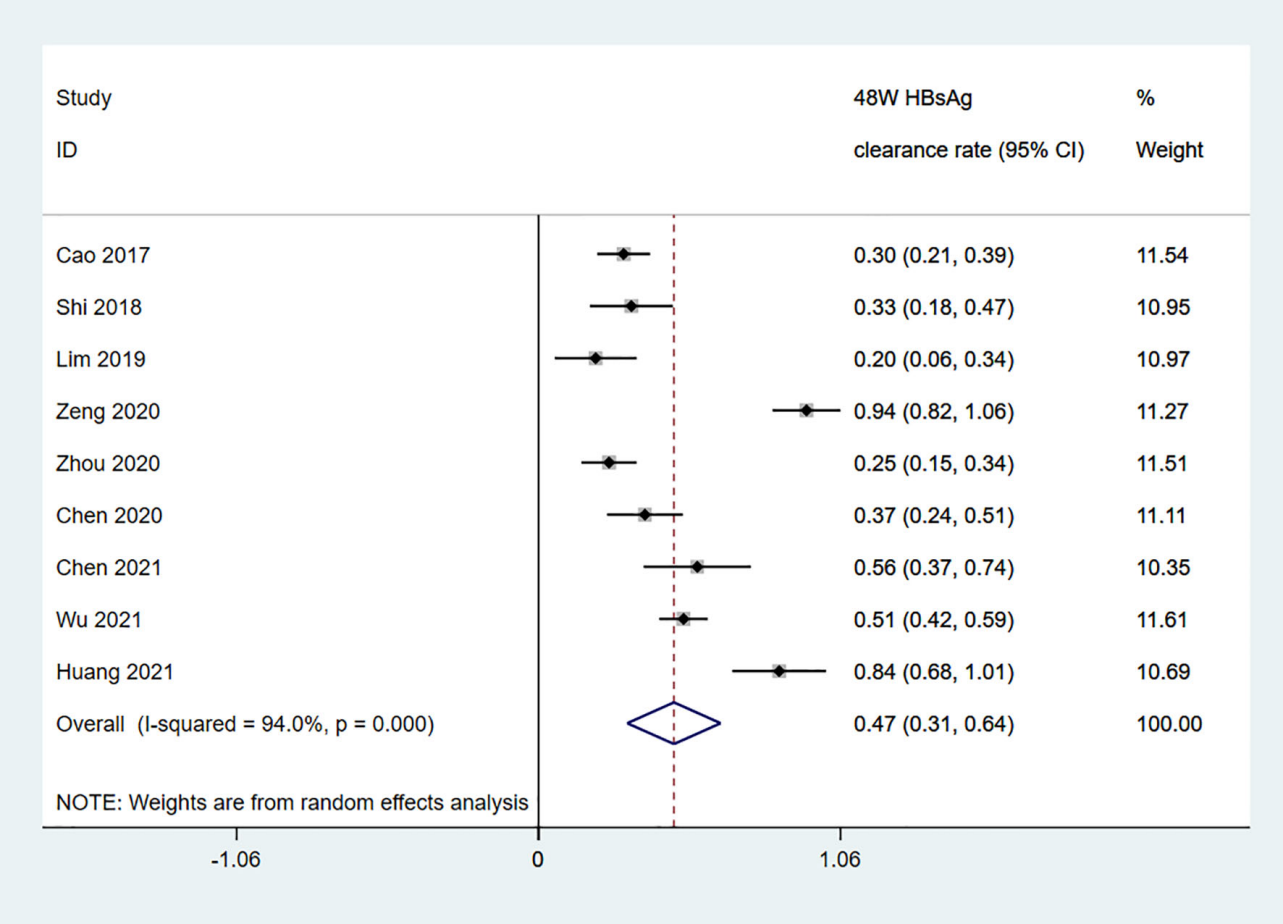
Meta-analysis of the pooled HBsAg clearance rate among IHCs after 48-W Peg-IFN treatment.

**Figure 3:**
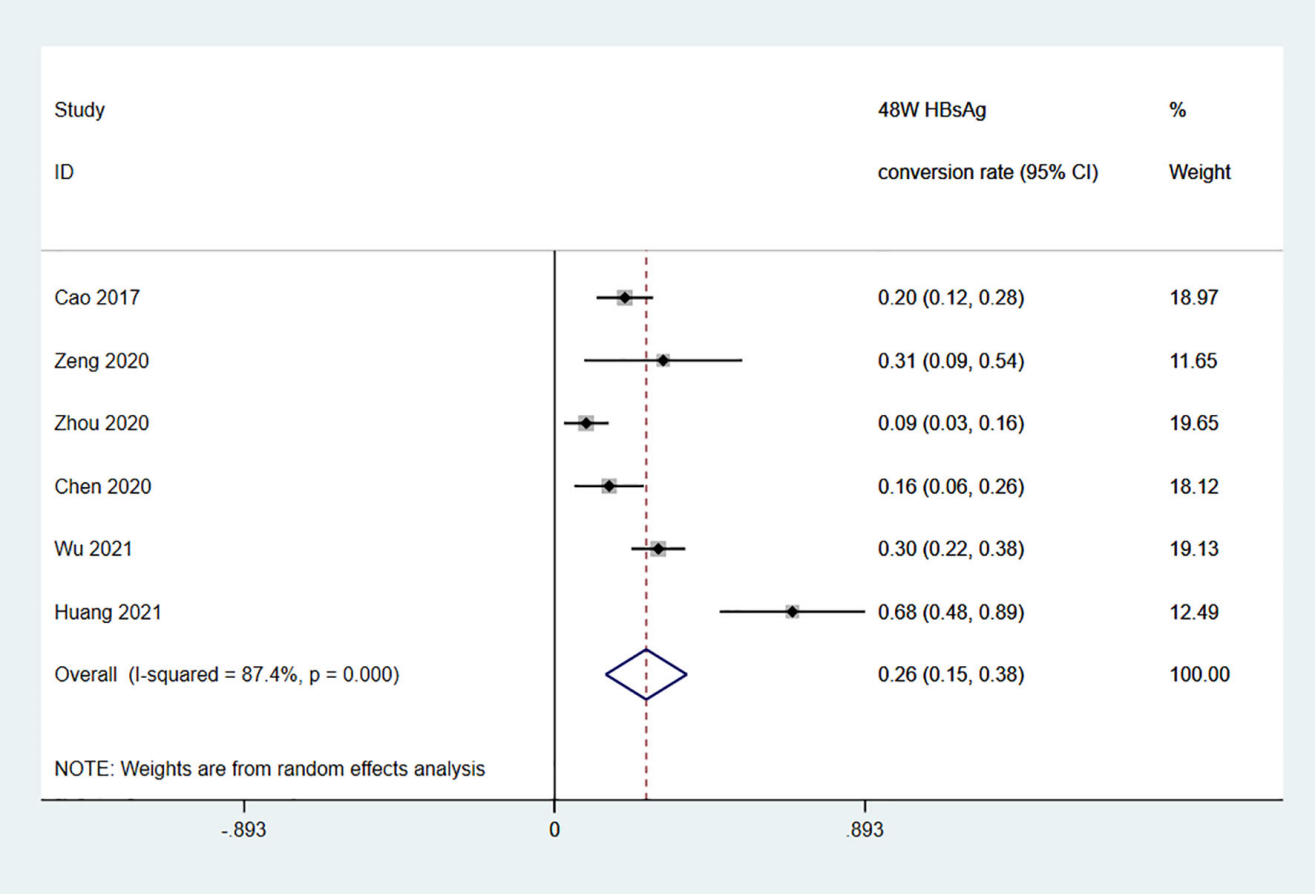
Meta-analysis of the HBsAg conversion rate among IHCs after 48-W Peg-IFN treatment.

**Figure 4:**
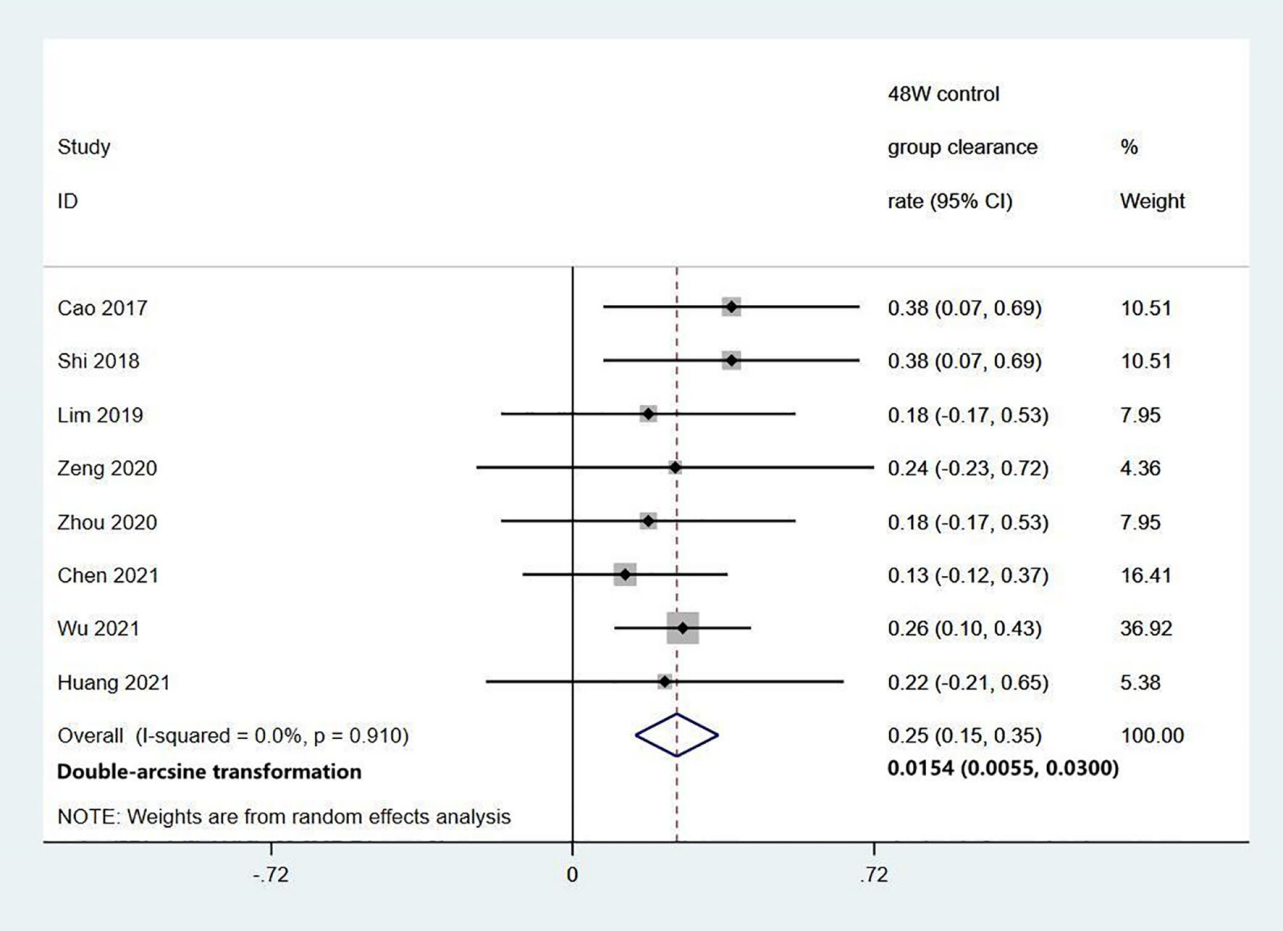
Meta-analysis of the HBsAg clearance rate in the control group.

Among the 11 studies on HBsAg clearance in IHCs after Peg-IFN treatment, the total sample size was 1029 patients, including 566 in the Peg-IFN group and 463 in the control group (NA: n=40, no treatment: n=423). Peg-IFN treatment lasted 24 to 96 weeks [24 weeks: n=1 (16); 48 weeks: n=9, 72 weeks: n=1 (12)]. For the nine studies with 48 weeks of Peg-IFN treatment, 199 of 488 patients achieved HBsAg clearance. Therefore, the overall HBsAg clearance rate was 47% (95% CI: 31% - 64%, *I^2^ = *94%, random-effects model, **Figure 2**). For publication bias, the shape of the funnel plot (**Figure S1**) was ideal, and Egger’s test showed *P*=0.55, suggesting no significant publication bias. Six of these nine studies reported HBsAg conversion after 48 weeks of Peg-IFN treatment. The overall conversion rate was 26% (92/391) (95% CI: 15% - 38%, **Figure 3**). In addition, eight studies (n=382) reported HBsAg clearance after 48 weeks of follow-up in the control group. Four patients achieved HBsAg clearance, and the overall clearance rate was 1.54% (95% CI: 0.56% - 3.00%, **Figure 4**). Egger’s test (*P*=0.9) suggested no significant publication bias. Eight studies reported HBsAg clearance at 48 weeks in the Peg-IFN group and the control group. The results showed that Peg-IFN treatment significantly increased the HBsAg clearance rate (relative risk (RR)=16.46, 95% CI: 7.60% - 35.66%, *P*<0.001, *I^2^ = *0%, **Figure S2**).

### HBsAg Clearance Rates for Different Baseline HBsAg Levels

**Figure 5:**
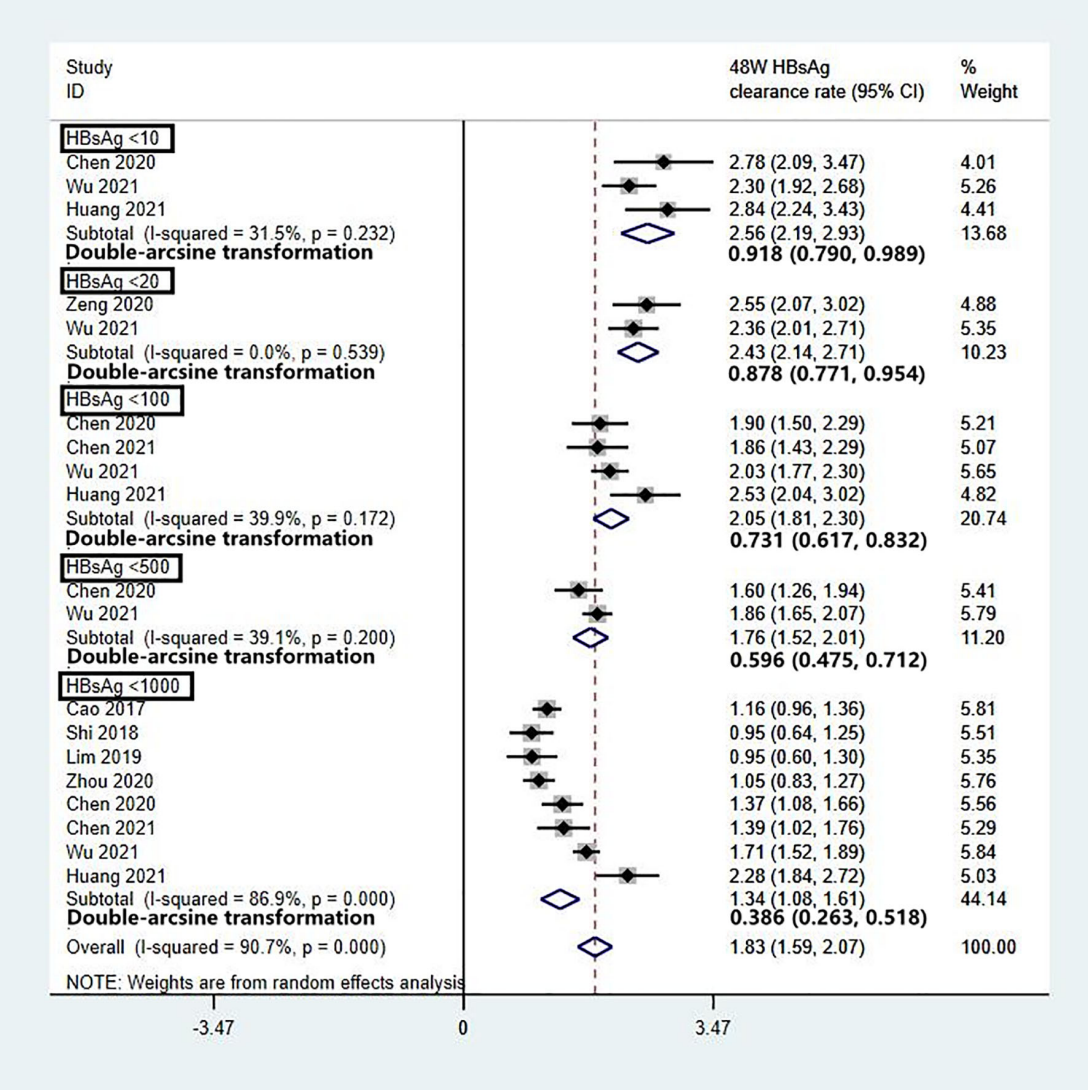
Subgroup analysis of the 48-W HBsAg clearance rate among different baseline HBsAg levels.

To evaluate the effect of baseline HBsAg on HBsAg clearance and reduce data heterogeneity, we analysed the data of patients who completed 48 weeks of Peg-IFN treatment, with stratification based on HBsAg level. The patients were divided into five groups based on their baseline HBsAg levels: <10 IU/mL, <20 IU/mL, <100 IU/mL, <500 IU/mL, and <1000 IU/mL. Two studies with baseline HBsAg levels <1500 IU/mL were excluded from the analysis due to data bias. The results showed that baseline HBsAg was inversely correlated with the clearance rate. After 48 weeks of Peg-IFN treatment, the HBsAg clearance rates were 92% (95% CI: 79% - 99%) for HBsAg <10 IU/mL, 88% (95% CI: 77% -95%) for <20 IU/mL, 73% (95% CI: 62% - 83%) for <100 IU/mL, 60% (95% CI: 48% - 71%) for <500 IU/mL, and 39% (95% CI: 25% - 53%) for <1000 IU/mL (**Figure 5**).

### HBsAg Clearance Rates in Different Treatment Periods

To evaluate the effect of Peg-IFN treatment courses on HBsAg clearance, we analysed the clearance rate among IHCs with baseline HBsAg <1000 IU/mL after Peg-IFN treatment. The HBsAg clearance rates were 29% (95% CI: 17% - 40%) after 24 weeks of treatment, 39% (95% CI: 25% - 53%) after 48 weeks of treatment, and 43% (95% CI: 35% - 50%) after 96 weeks of treatment (**Figure S3**), suggesting that HBsAg clearance increased with longer Peg-IFN treatment, although the difference did not reach statistical significance (*P*=0.5). This finding may be related to the small sample sizes and significant heterogeneity in the 24-week and 48-week groups.

### Additional Results

In addition to baseline HBsAg, several studies reported that post-Peg-IFN treatment HBsAg and ALT were also strong predictors of HBsAg clearance. Five studies showed that decreased HBsAg after 12 or 24 weeks of Peg-IFN treatment and elevated ALT after 4 or 12 weeks of Peg-IFN treatment were effective predictors of HBsAg clearance (11, 12, 15, 18, 19). One study reported that hepatitis B vaccination contributed to HBsAg clearance (14), and one study reported that baseline HBV DNA <20 IU/mL was a favourable factor for HBsAg clearance (15).

Most studies reported adverse side effects during treatment, including neutropenia, thrombocytopenia, pyrexia, fatigue, hair loss, weight loss, and rash, most of which were mild and resolved after symptomatic care. In addition, a few patients experienced thyroid dysfunction, anxiety disorder, and lipsotrichia, and some patients required treatment suspension or dose adjustment but were able to resume and complete the Peg-IFN treatment course, which is similar to observations during previous CHB treatment. More than half of the patients had increased ALT levels during treatment, which was a strong predictor of HBsAg clearance. Drugs can be used to reduce enzymes and protect the liver. Normalization of ALT levels coincided with HBsAg clearance and was maintained during the follow-up.

## Discussion

For CHB patients, HBsAg clearance or conversion is regarded as a “functional cure” in antiviral therapy (2, 3, 22). With HBsAg clearance, patients can safely discontinue treatment with optimal long-term outcomes (23, 24). However, the HBsAg clearance rate is still low (3% to 7%) in CHB patients after 48 weeks of Peg-IFN treatment (25, 26). In this meta-analysis, we focused on the effect of the same treatment and treatment course in IHCs and found that the HBsAg clearance rate increased by more than 10 times to 47%. In the Asia-Pacific region, especially in China, a very large IHC community can benefit from short-term Peg-IFN treatment, suggesting that IHCs should be eligible for antiviral therapy.

This meta-analysis of 1029 IHCs from 11 studies showed that after 48 weeks of Peg-IFN treatment, the overall clearance rate was relatively high at 47% among 488 patients, and the HBsAg conversion rate was 26%. In the control group (n=382), the overall clearance rate was only 1.54%, which was markedly lower than that in the Peg-IFN group, suggesting that IHCs may be clinically cured and that Peg-IFN treatment is far more effective than NA treatment in achieving this goal.

The clearance rate ranged from 20% to 94% across the studies due to different baseline HBsAg levels, Peg-IFN treatment courses, and sample sizes. To minimize data heterogeneity, we first analysed factors affecting HBsAg clearance in IHCs who completed 48 weeks of Peg-IFN treatment and found that baseline HBsAg was inversely correlated with the clearance rate, which was 92% among IHCs with baseline HBsAg levels <10 IU/mL. Next, we analysed the clearance rate after different Peg-IFN treatment courses in patients with baseline HBsAg levels <1000 IU/mL and found that the HBsAg clearance rates were 29% after 24 weeks of treatment, 39% after 48 weeks of treatment, and 43% after 96 weeks of treatment. These data indicate that a low baseline HBsAg level and longer Peg-IFN treatment are favourable factors for HBsAg clearance with practical value for the clinical treatment of IHCs along with the predictive value of post-Peg-IFN treatment HBsAg and ALT. While the overall HBsAg clearance rate is substantially higher among IHCs than among CHB patients, more precise planning can enable targeted treatment to help IHCs achieve a clinical cure and an optimal outcome as early as possible and at a lower cost. Patients who do not respond to Peg-IFN treatment can discontinue treatment early to prevent adverse drug reactions, which also has pharmacoeconomic value.

This study has some limitations. First, because current Chinese and international guidelines do not recommend treatment for IHCs, few prospective, multicentre cohort studies have been conducted to investigate posttreatment HBsAg clearance in IHCs, and the sample sizes in available studies are usually small. Second, all studies included in this meta-analysis were conducted in the Asia-Pacific region, without any study from Europe or the US, which is due to the regional deviation in the hepatitis B prevalence. The European and American hepatitis B guidelines do not recommend treatment for IHCs. This recommendation is primarily based on studies in Caucasian populations in Europe and the US showing that for IHCs, the HBsAg conversion rate is 15% to 45% over a 10-year follow-up period, without any significant increase in the incidence of HCC compared to that for the general population (27). However, studies in the Asia-Pacific region have reached different conclusions, showing that for IHCs, the HBsAg clearance rate is only 0.5% to 1% over a long follow-up (5). In a long-term follow-up study with 1932 patients, the mortality rates of HCC and liver disease were 4.6-fold and 2.1-fold higher in IHCs than in HBsAg(-) individuals, respectively (6). These data from Asian populations suggest that for IHCs, withholding treatment causes more harm than good, mainly because in Asian populations, HBV infection often occurs in young people, and patients usually have a long disease course by the CHB stage. Therefore, treating IHC patients in the Asia-Pacific region is important. Third, the follow-up time was short in clinically cured IHCs after treatment, and long-term outcome data are lacking. Fourth, sample sizes vary across studies due to different geographic regions and subjects. Therefore, the results of this meta-analysis have some inherent heterogeneities.

In short, this meta-analysis shows that IHCs may achieve a high HBsAg clearance rate (47%) after Peg-IFN treatment. Given the very large IHC population and the high response rate, IHCs should be treated with antiviral therapy, as they have the potential to be clinically cured.

## Data Availability Statement

The original contributions presented in the study are included in the article/**Supplementary Material**. Further inquiries can be directed to the corresponding authors.

## Author Contributions

AS, XL, and XC conceived and designed the protocol and study. JL and SR identified studies to be screened. ZC and HL identified studies for eligibility, extracted data, and assessed the methodological quality of the included studies. AS performed the analysis with assistance from SZ, ZH, CS, and XC. All authors contributed to the article and approved the submitted version.

## Funding

This work was supported by the Thirteenth Five-Year Major Science and Technology Projects (2017ZX10202201, 2017ZX10201201-001-008, 2017ZX10302201-004-003, 2017ZX10202202-005-010), the Capital Health Research and Development Projects (2020-1-2181), the Beijing Municipal Administration of Hospitals Clinical Medicine Development of Special Funding Support (ZYLX202125), and the Key R&D and Transformation Plan in Qinghai Province (No. 2017-SF-159).

## Conflict of Interest

The authors declare that the research was conducted in the absence of any commercial or financial relationships that could be construed as a potential conflict of interest.

## Publisher’s Note

All claims expressed in this article are solely those of the authors and do not necessarily represent those of their affiliated organizations, or those of the publisher, the editors and the reviewers. Any product that may be evaluated in this article, or claim that may be made by its manufacturer, is not guaranteed or endorsed by the publisher.
