# Supplementary material for: Pegylated Interferon Treatment for the Effective Clearance of Hepatitis B Surface Antigen in Inactive HBsAg Carriers: A Meta-Analysis
Source: Front Immunol. 2021 Nov 4;12:779347. doi: 10.3389/fimmu.2021.779347 (PMC8600041; doi:10.3389/fimmu.2021.779347)
Supplement: Supplementary file 1 [file DataSheet_1.docx]

| **Supplementary Material**  **Table S1. Summary of critical appraisal of included studies using the Newcastle-Ottawa Quality Assessment Scale.**  **Study** | **Selection** | | | | **Comparability** | **Outcome** | | | **Total**  **score** |
| --- | --- | --- | --- | --- | --- | --- | --- | --- | --- |
|  | **Representativeness** | **Selection of non-exposed from the same community** | **Ascertainment of exposure** | **Demonstration outcome not present at start** | **Comparability**  **of cohorts** | **Assessment of outcome** | **Follow**  **up long enough** | **Adequate follow-up rate (≥80%)** |  |
| Cao 2017 | **✓** | **✓** | **✓** | **✓** | **✓✓** | **✓** | **✓** | **✓** | 9 |
| Li 2016 | **✓** | **✓** | **✓** | **✓** | **✓×** | **✓** | **✓** | **✓** | 8 |
| Lim 2019 | **✓** | **✓** | **✓** | **✓** | **✓×** | **✓** | **✓** | **✓** | 8 |
| Zeng 2020 | **✓** | **✓** | **✓** | **✓** | **✓×** | **✓** | **✓** | **✓** | 9 |
| Wu 2021 | **✓** | **✓** | **✓** | **✓** | **✓✓** | **✓** | **✓** | **✓** | 9 |
| Zhao 2020 | **✓** | **✓** | **✓** | **✓** | **✓×** | **✓** | **×** | **✓** | 7 |
| Shi 2018 | **✓** | **✓** | **✓** | **✓** | **××** | **✓** | **✓** | **✓** | 7 |
| Zhou 2020 | **✓** | **✓** | **✓** | **✓** | **✓✓** | **✓** | **✓** | **✓** | 9 |
| Chen 2020 | **✓** | **×** | **✓** | **✓** | **××** | **✓** | **✓** | **✓** | 6 |
| Chen 2021 | **✓** | **✓** | **✓** | **✓** | **✓✓** | **✓** | **✓** | **✓** | 9 |
| Huang 2021 | **✓** | **✓** | **✓** | **✓** | **✓✓** | **✓** | **✓** | **✓** | 9 |

**
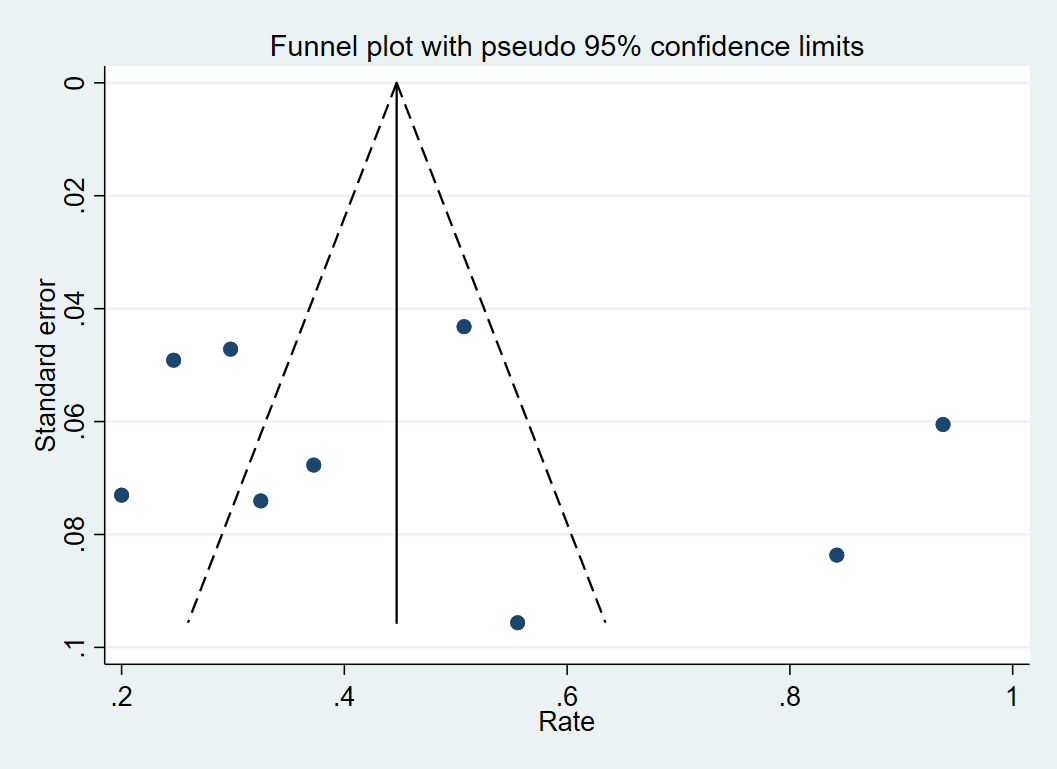
**

**Figure S1. Funnel plot of pooled recurrence rate meta-analysis.**

**
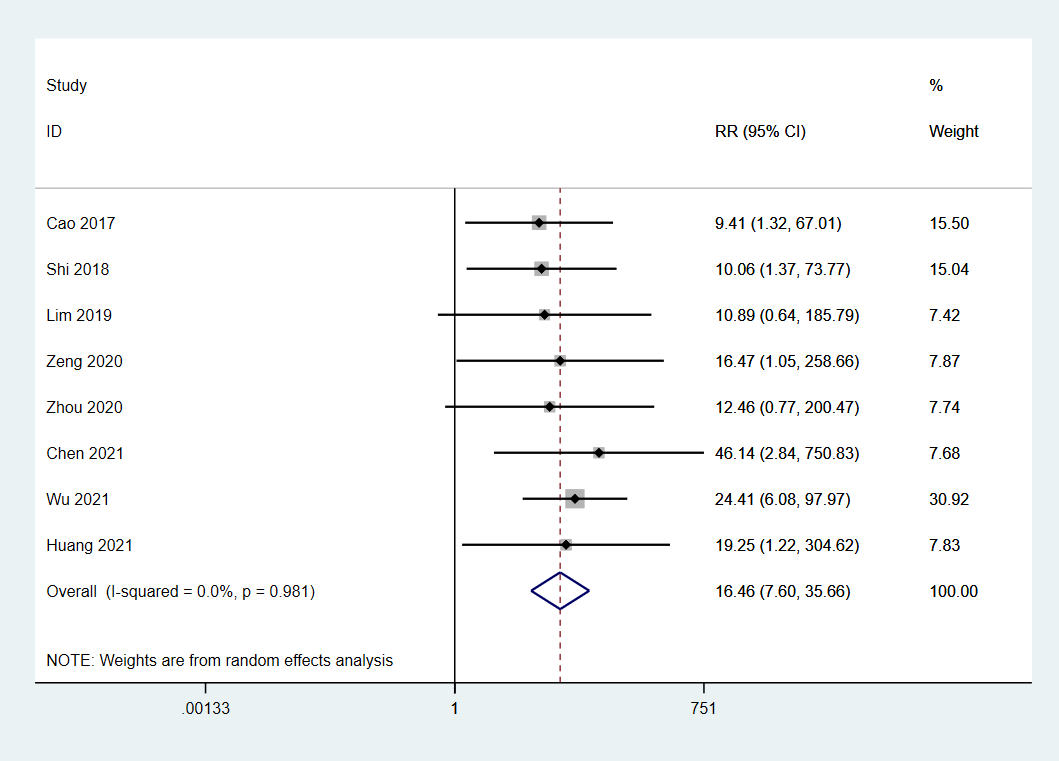
**

**Figure S2. Meta-analysis of HBsAg clearance rate in Peg-IFN group vs. control group**

**
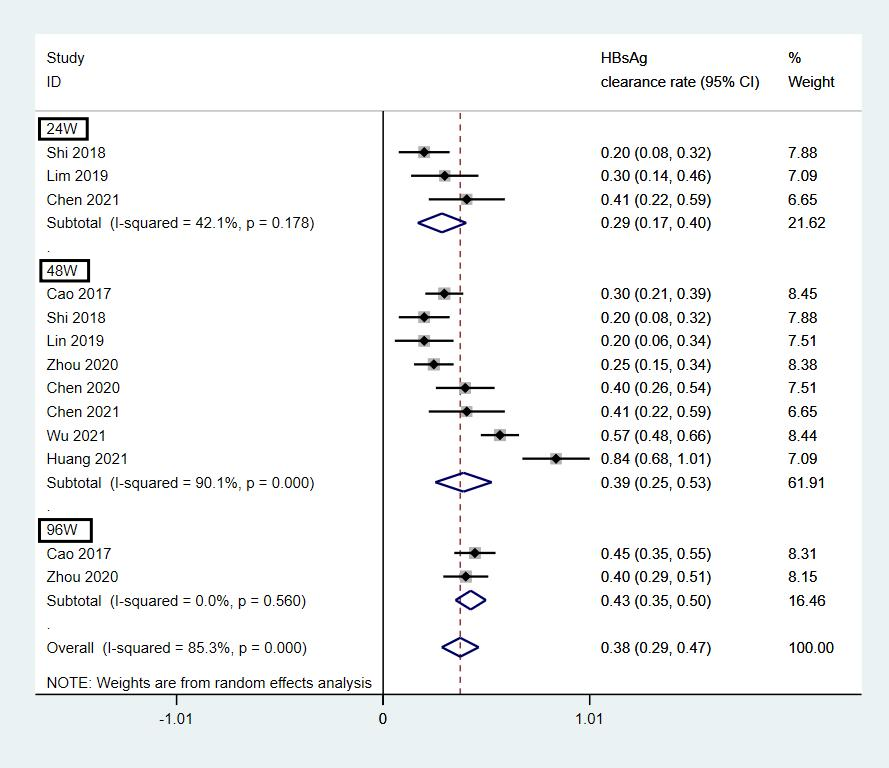
**

**Figure S3. Subgroup analysis of HBsAg clearance rate in baseline HBsAg 1000 IU/mL patients among different treatment period**
